# NETosis provides the link between activation of neutrophils on hemodialysis membrane and comorbidities in dialyzed patients

**DOI:** 10.1007/s00011-016-1010-6

**Published:** 2016-11-24

**Authors:** Marie Korabecna, Vladimir Tesar

**Affiliations:** 1 Department of Biology and Medical Genetics, First Faculty of Medicine, Charles University and General University Hospital in Prague, Albertov 4, 128 00 Prague, Czech Republic; 2 Department of Nephrology, First Faculty of Medicine, Charles University and General University Hospital in Prague, U Nemocnice 2, 128 08 Prague, Czech Republic

**Keywords:** Neutrophil extracellular trap, NETosis, Cell-free DNA, Hemodialysis, Diabetes, Thrombosis, Atherosclerosis

## Abstract

**Introduction:**

Neutrophil extracellular traps (NETs) are formed by activated neutrophils during the process of NETosis in which the nuclear material is released into extracellular space, including DNA molecules, citrullinated histones, and neutrophil granule enzymes, such as elastase. This material forms networks that are able not only to physically entrap bacteria but also to provide elevated concentration of bactericidal components. Over the last years, it has become clear that NETs can also be formed under numerous sterile inflammatory conditions, i.e., thrombosis, cancer, SLE, atherosclerosis, and diabetes.

**Method:**

We reviewed studies published until July 2016 to find possible associations between elevated cell-free DNA levels in dialyzed patients and the process of NETosis and its consequences.

**Results:**

The process of NETosis, its elevated activation, or impaired clearance provides the link between clinical conditions and elevated levels of cell-free DNA found in plasma after the hemodialytic procedure which itself is able to activate neutrophils via platelets and ROS formation. NETs stimulate thrombosis and endothelial damage, and their formation may contribute to the development of spectrum of comorbidities described in dialyzed patients.

**Conclusion:**

The study of plasma cell-free DNA levels together with markers of NETosis could contribute to the evaluation of the influence of hemodialysis on the immune system of patients.

## Introduction

The fact that neutrophils can form extracellular traps (neutrophil extracellular traps—NETs) containing DNA to kill bacteria was first observed and described in 2004 by Brinkmann and colleagues [1]. The term “NETosis” has been coined in 2007 by Steinberg and Grinstein [2] to denote the neutrophil cell death in consequence of this newly described mechanism of innate immune response primarily recognized as defense against microbial infection.

Since the discoveries, the process of NETosis has been explored in greater detail. It has been found that it can be activated not only by bacterial infection but also under sterile conditions associated with thrombosis, cancer, atherosclerosis, and diabetes [3]. The first results documenting the role of NETosis in hemodialysed (HD) patients were published [4, 5] as well as articles elucidating the functional connections between NETosis and comorbidities frequently found in HD patients, such as diabetes, chronic inflammation, major adverse cardiovascular events, autoimmune diseases, and cancer [4, 6–13].

We studied the cell-free DNA (cfDNA) levels in plasma in HD patients before and after a hemodialysis session [14]. In accordance with others similar studies [15, 16], we found elevated cfDNA concentrations in plasma after the procedure. In patients undergoing the peritoneal dialysis, the relationship between the cfDNA concentration in dialysate and the overall length of therapy has been found [14]. The recent knowledge with regard to the process of NETosis elucidates these previously obtained results.

In present minireview, our attempt is to summarize what is recently known about the NETosis generally and about the connection between this process; hemodialysis and comorbidities in HD patients with the goal to stimulate intensive research of this interesting phenomenon with multiple potential clinical applications not exclusively for HD patients.

## General characteristics of NETosis

### Morphological and molecular features of NETosis

NETosis is described as a new type of a programmed cell death following the multi-step highly coordinated scenario based on histone citrullination, chromatin decondensation, migration of elastase and other neutrophil granule enzymes into the nucleus, disintegration of nuclear membranes, and the release of neutrophil extracellular traps (NETs) containing DNA, citrullinated histones, and enzymes of neutrophil granules into extracellular space. NETs are characterized by specific ultrastructure. They are formed by chromatin filaments having 15–17 nm in diameter [1]. The filaments can be organized into cloud-like structures and occupy a 10–15-fold larger area than the cell from which they originated [17]. The NETs contain not only DNA and histones (histones account for 70% of their protein content), but also globular structures of about 50 nm in diameter are observed inside the NETs. Such structures represent the source of numerous components of neutrophil granules, such as neutrophil elastase (NE), myeloperoxidase (MPO), cathepsin G, proteinase 3, BPI (cationic bactericidal/permeability increasing protein), calgranulin, α-defensins, lactoferrin, and a fragment of the protein cathelicidin hCAP18—the peptide LL-37, and pentraxin PTX3, matrix-metalloproteinase 9 (MMP-9), and peptidoglycan recognition protein-S (PGRP-S) [17–20].

Different types of cell death are usually defined by morphological criteria (Table 1) [21]. Recently, it has become clear that all these modes of cell death are interconnected at the molecular level by the network of overlapping signaling pathways; therefore, it has been proposed to use instead the terms “apoptosis”, “autophagy”, and “necrosis”; the term “regulated necroptosis” for all cases of cell death is not caused accidentally by external factors [22]. As the extracellular traps containing chromatin may be extruded also by eosinophils, mast cells, and monocytes/macrophages, the term ETosis (concerning Extracellular Traps formation) has been proposed to denote this process [17].

**Table 1:** Classification of distinct types of cell death according to the recommendations of the Nomenclature Committee on Cell Death 2009 [21] and to recent knowledge of interconnected signaling pathways [22]

Cell death type			Main characteristics
Regulated necroptosis [22]	Apoptosis		Rounding-up of the cell Nuclear fragmentation DNA fragmentation Plasma membrane blebbing and rupture Activation of caspases Apoptotic bodies formation Minor modification of cytoplasmic organelles ROS overgeneration Engulfment by resident phagocytes in vivo
	Autophagy		Lack of chromatin condensation Digestion of cellular organelles—accumulation of autophagic vacuoles Massive vacuolization of the cytoplasm
	Necrosis	Regulated necrosis = necroptosis (including NETosis)	Cytoplasmic swelling (oncosis) Rupture of plasma membrane and spilling of intracellular content ROS overgeneration
		Accidental necrosis	Caused by external factors

The methods allowing the quantification of NETosis-specific markers are elaborated and the first reports concerning their use appeared [23, 24]. Unfortunately, some methods are based on artificial stimulation of NETosis in isolated cell population in vitro (e.g., stimulation of cells with phorbol myristate [23]) and they are not applicable to analysis of plasma samples. ELISA with anti-MPO antibodies seems to be most promising methods for analysis of cell-free samples followed by flow cytometry with fluorescent dye SYTOX^®^ Green which label DNA but do not enter intact cells [24].

### Activation of NETosis

The molecular mechanisms activating the process of NETosis are not fully understood. It is clear that the ROS play one of the most important roles in this event. The best inducer of NEtosis in vitro is phorbol 12-myristate 13-acetate (PMA) which belongs to strong inducers of ROS generation [25]. ROS are probably needed for oxidative modifications of macromolecules including DNA. Activation of neutrophils involves the activity of NADPH oxidase complex on the cytoplasmic membrane and on the membrane of neutrophil granules. Neutrophils without the ability to form NETs are found in patients with chronic granulomatous disease (CGD) caused by mutation in a gene for NAPH oxidase subunit [26]. Signal transduction from the receptors to NADPH oxidase is dependent on Raf/MEK/ERK-signaling pathway [27] and also on Rac-2-mediated pathway [28].

### Molecular events associated with NETosis

During the first hour after activation of NETosis, the nucleus loses its lobules, the membranes of nuclear envelope separate, and later, they form vesicles, chromatin decondenses, and the neutrophil granules disintegrate. Then, nucleoplasm and cytoplasm merge, cytoplasmic membrane is broken, and the content of the cell is released into extracellular space to form the NET [17]. Enzymes of primary (azurophilic) granules of neutrophils—neutrophil elastase (NE) and myeloperoxidase (MPO)—participate in the process of chromatin decondensation. After the initiation of NETosis, they are transported in granules into the nucleus, where NE cleaves the linker histone H1 and modifies core histones. Mice deficient in this enzyme are not able to produce NETs [29]. The process of NET formation is also impaired in patients with mutations in the *MPO* gene [30]. The enzyme peptidylarginine deiminase 4 (PAD 4) catalyzes deamination of arginine residues yielding citrullines in the core histones. Citrullination of histones is found in decondensed chromatin [31–33]. The PAD4 activity and the presence of citrullinated histones are regarded as very useful specific markers of NETosis [34], because PAD4 is suppressed after induction of apoptosis [32].

The modification of the process of NETosis which does not lead to the cell death has also been described. This process is referred as *vital NETosis* in contrast to the above-mentioned process of *suicidal NETosis* [35]. Vital NETosis may be induced in different ROS-independent ways, and NETs are then excreted from vesicles formed within cytoplasm and fusing with cell membrane. Neutrophils after vital NETosis despite the complete loss of the nucleus retain their biological functions, such as chemotaxis and phagocytosis [36–38]. Among the stimuli leading to activation of vital NETosis, the microbial ligands, toll-like receptors (TLR 2 and 4), and platelets were recognized as the most important players [36–38].

ROS-dependent formation of NETs from mitochondrial DNA but not nuclear DNA by viable neutrophils has also been observed [39]. Neutrophil extracellular traps formed after major trauma and subsequent surgery contain mtDNA [40].

Antimicrobial activity of NETs was explored widely. The fact that NETs avoid the dissemination of bacteria in the body has been proven in experiments employing artificial bacterial infection of animals and their treatment with DNases [37]. The study of microbicidal properties of NETs has not provided clear results. Microbicidal activity of histones was reported [3], but DNA is thought to be the major antimicrobial component of NETs [41]. On the contrary, it has been reported that the bacteria captured in NETs and subsequently released using DNase preserved full viability [42]. Blood plasma contains large amount of protease inhibitors which may effectively inactivate the NET components, and the experiments demonstrating the microbicidal activity of NETs were performed in a serum-free medium [35]. Therefore, further studies are needed for elucidation of all NET functions.

NETs with their complex composition may be considered as typical representatives of alarmins. Alarmins were defined as molecules providing a danger signal associated not only with infections but also with tissue damage. According to the definition [43], alarmins can be released passively from dying cells or actively from stimulated immune cells, and they can diffuse and induce the immune response, including the sterile inflammation and tissue reparation. DNA belongs to the strong activators of immune system. Different immune cells are equipped by endosomal and cytosolic receptors for recognition of autologous DNA molecules. Plasmacytoid dendritic cells contain, e.g., the endosomal receptor TLR9, and monocytes/macrophages have numerous other cytosolic receptors. The recognition of DNA by these receptors activates the signal pathways leading to synthesis of interferons of type I (I IFN) and a number of pro-inflammatory cytokines. Complexes containing DNA associated with the peptide LL-37 which also belongs to the NET components are endocytosed by TLR9 bearing endosomes, and the regulatory pathways stimulates the I IFN synthesis (reviewed in detail in [17]). In opposite way, the inflammatory cytokines may trigger the process of NETosis similarly to microbial liposacharides, immune complexes, and autoantibodies [6].

### NET clearance

The clearance of NETs is not fully understood. *In vitro*, the NETs are degraded by the addition of DNase 1 [1]. The role of DNase 1 in NET degradation has also been proven in vivo [44, 45]. It is known that circulating DNA has a short half-life (10–15 min) [16, 46]. Impaired process of NET clearance is considered as an important pre-requisite in the development of autoimmune diseases, namely, systemic lupus erythematosus (SLE) has been explored with regard to involvement of NETs in its pathogenesis—the antibodies against all components of NETs were found in the blood of SLE patients [17].

## NETosis and dialysis

### NETosis and hemodialysis

The levels of cell-free DNA (cfDNA) were studied before the start and after the completion of a hemodialysis procedure. Irrespective of the type of hemodialysis membrane, the mutually confirming results were obtained by independent studies [14–16]. All studies reported the elevation of concentrations of cfDNA in patients’ plasma after the procedure, but it has been difficult to interpret such results. Atamaniuk et al. [15] correlated the levels of cfDNA in HD patients with levels of markers of apoptosis in their plasma and concluded that there is an increased level of apoptosis in blood of patients during hemodialysis. On the contrary, we were not able to correlate the elevated levels of cfDNA with decreased numbers of leucocytes in patient’s blood after the procedure [47]. Simultaneously, the fact that the hemodialysis activates neutrophils, and consequently, the elevated production of microparticles from neutrophils and platelets as non-specific markers of neutrophil activation during dialysis-induced inflammation has been reported [48].

Tovbin et al. [49] regarded cfDNA as an integrative marker of tissue damage. Their study evaluated postdialysis cfDNA levels as an independent predictor of all-cause mortality in HD patients.

Jeong da Wun et al. [5] studied the levels of cfDNA in HD patients with diabetes and with cardiovascular diseases. They reported increased levels of cfDNA in these patients in comparison with HD patients without such comorbidities. The levels of cfDNA correlated positively with the counts of white blood cells. The researchers concluded that uncontrolled hypertension and poor glycemic control are independent determinants for the elevated cfDNA. As McGuire et al. [50] demonstrated that cfDNA levels are not influenced by renal impairment but do reflect endothelial dysfunction in patients with chronic kidney disease (CKD), and the study by Jeong da Wun et al. [5] stated that cfDNA might be a marker of vascular injury rather than pro-inflammatory condition in HD patients.

In the opposite way, cfDNA in plasma of HD patients may stimulate the production of IL-6 as demonstrated in an experimental study by Atamaniuk et al. [51]. The researchers studied the effects of plasma collected from HD patients on the activity of monocytes in vitro. Plasma from HD patients but not from healthy controls or DNase I-treated HD plasma induced IL-6 production from monocytes. The study concluded that the cfDNA contained in plasma of HD patients selectively stimulates the production of the pro-inflammatory cytokine interleukin IL-6 in human monocytes. Jeong et al. [4] in 2016 as the first researchers connected all the known facts and took the process of NETosis in account when studying HD patients and their risk of major adverse cardiovascular events (MACE).

The researchers measured NET formation markers, including DNA-histone complexes and cfDNA, neutrophil elastase, and IL-6 in HD patients and investigated their potential as predictors of cardiovascular risk and mortality. They also focused on the exploration of the role of uremic toxins with regard to their potential to stimulate the production of DNA-histone complexes and cfDNA in peripheral neutrophils from normal volunteers. This study provided the evidence that uremic toxins induce DNA-histone complex formation and the elevated levels of cfDNA in an in vitro experiment. The levels of DNA-histone complexes were evaluated as a significant independent predictor of major adverse cardiovascular events (MACE) in HD patients.

The DNA-histone complexes were regarded as markers of NET formation associated with inflammatory conditions in this study. Inflammatory status of studied HD patients was confirmed by elevated levels of inflammatory cytokine IL-6.

### NETosis and peritoneal dialysis

In our study [14], we detected cfDNA in overnight effluents of patients undergoing peritoneal dialysis (PD). The concentrations of cfDNA in these effluents correlated inversely with the duration of PD treatment. The activation of the process of NETosis through the effects of glucose degradation products (GDP) in peritoneal dialysis solutions gives the clue to the explanation of this phenomenon. Chronic exposure of the peritoneal membrane to GDPs and dextrose leads to the formation of advanced glycosylation end products (AGEs) that bind to receptors for AGE (RAGE). It leads to the generation of several cytokines stimulating inflammatory changes in the peritoneal space [52]. The elevated level of glucose itself is able to contribute to the enhanced activation of NETosis as has been demonstrated experimentally with neutrophils from diabetic humans and mice which were primed to produce NETs [8]. AGEs may mediate the activation of NADPH oxidase and predispose neutrophils to NETosis [53]. Bansal et al. [54] investigated the effect of AGE on reactive oxygen and nitrogen species generation and subsequent oxidative stress in neutrophils. This study provided evidence that AGEs may play a key role in the induction of oxidative stress in neutrophils which is the pre-requisite for activation of NETosis. Neutrophils from non-diabetic individuals are prone to NETosis when exposed to high glucose [9]. Osmotic stress also induces the process of NETosis [55].

NETosis in peritoneal cavity in the response to the treatment by peritoneal dialysis has not been studied yet. It seems that the research focused on this problem could provide new interesting insights into the complex regulatory network associated with biocompatibility of PD solutions.

## NETosis and frequent comorbidities in hemodialyzed patients

The elevated levels of NET formation or their persistence have been demonstrated in clinical disorders which frequently occur in HD patients—cardiovascular diseases, including atherosclerosis and thrombosis [10], diabetes II [8], autoimmune diseases, such as systemic lupus erythematosus [11], and wide spectrum of oncologic diseases [13].

### Cardiovascular diseases

Cardiovascular disease belongs to the major causes of morbidity and mortality in HD patients with end-stage renal disease [56]. It has been shown that the presence of cfDNA together with platelet–neutrophil interactions may promote microvascular thrombosis [6]. Increased levels of cfDNA were reported in patients with deep vein thrombosis, and NET formation was detected as essential for development of venous thrombosis [12, 57]. Multiple mechanisms are discussed as being potentially involved in the pathogenic activation of coagulation in the presence of NETs. These mechanisms are based on extracellular release of tissue factors, activity of polyanionic surface of NETs, or proteolytic cleavage of tissue factors and serpins by elastase released by neutrophils during NETosis [6].

The complex role of NETosis in pathogenesis of atherosclerosis has been partially elucidated. Cholesterol crystals activate the release of NETs, which are able to stimulate macrophages to release the cytokines. In this manner, the immune cell recruitment into atherotic plaques is intensified [58]. NETosis activated by lipopolysacharides of bacterial walls also explains the old theory postulating that the presence of periodontal pathogens in blood may start the atherosclerotic changes [59]. Endothelial dysfunction is regarded as crucial in pathogenesis of atherosclerosis. Activated endothelial cells are able to stimulate NETosis, but on the other hand, they are themselves damaged by NETs [60]. In severe glomerulonephritis, NET-related extracellular histones promote vascular necrosis [61].

### Diabetes

Exacerbated NETosis is known as factor complicating wound healing in diabetic patients [8]. The high glucose levels contribute to the activation of NETosis [8, 9]. NETosis can play role in the onset of type I diabetes, where the components of NETs activate autoimmune processes [62].

### Autoimmune diseases

NETs were detected in affected kidneys in patients with SLE [63], in autoimmune small-vessel vasculitis [64], and in vasculitis lesions associated with anti-neutrophil cytoplasmic antibodies (ANCA) [65]. The presence of NETs in impaired tissues provides the evidence that neutrophils are included in pathogenesis, but it is not clear whether they initiate the glomerular or vascular tissue damage or whether they only contribute to acceleration of an independently activated process [34]. In patients with SLE, the imbalance between NET formation and NET clearance was reported. Increased number of dying lymphocytes in the blood of SLE patients correlates with increased titers of anti-DNA autoantibodies. Simultaneously, SLE sera have reduced capacity to degrade NETs [34].

In rheumatoid arthritis, the autoantibodies to a citrullinated protein fillagrin are detected. Many patients also express the autoantibodies to histones. Therefore, it is reasonable to suppose that the NETosis is also involved in the pathogenesis of this disease [34].

### Oncologic diseases

The study of the roles of NETosis in cancerogenesis represents an exciting newly emerging topic with many practical implications. It has been reviewed in detail by Cools-Lartigue et al. [13]. Neutrophils were recognized as important components of the tumor-associated inflammatory cell infiltrates [66, 67]. Their roles in tumor biology are intensively studied, and both pro- and anti-tumorigenic properties are found [68]. The role of cytokines as tumor necrosis factor alpha (TNF-a) and interleukin 8 (IL-8) in the facilitation of the process of NET formation was proven. Both these cytokines are released by numerous primary tumor types [13]. The results of the first performed studies suggest a possible association between intra-tumoral NET deposition and tumor progression [66, 67]. The ability of tumor cells to predispose circulating neutrophils to produce NETs was demonstrated in different tumor types [69]. In the context of carcinogenesis, it is hypothesized that NETs may play a similar role as in limitation of bacterial infection—they provide high local concentrations of biologically active proteins which are able to promote proliferation and inhibit apoptosis. With regard to metastasis, the NETs can entrap circulating tumor cells. The NET component—matrix-metalloproteinase 9 (MMP-9)—participates in the degradation of the extracellular matrix, and it is involved in tissue remodeling, angiogenesis, and tumor progression [70]. Neutrophil elastase contained in NETs may also participate in extracellular matrix degradation (especially in the cleavage of elastin) and tissue remodeling to facilitate the tumor growth [71]. Another component of NETs—cathepsin G—facilitates angiogenesis and tumor cell dissemination [72]. NETs are recognized by leukocytes including macrophages and dendritic cells [13], and in this manner, the persistent inflammatory state may be established. This way, NET deposition could create a “pre-metastatic niche”. It is known that systemic sepsis promotes the development of metastasis and administration of inhibitors of NET formation was able to attenuate this process [13]. The study by Cools-Lartigue et al. [66] demonstrated that disruption of NETs using either DNase or neutrophil elastase inhibitors avoids cell adhesion and metastasis formation. This study highlighted NETs as possible therapeutic targets. Treatment with DNase seems to be clinically safe as reported in patients with SLE [73].

## Future perspectives of NETosis exploration in dialyzed patients

**Fig. 1:**
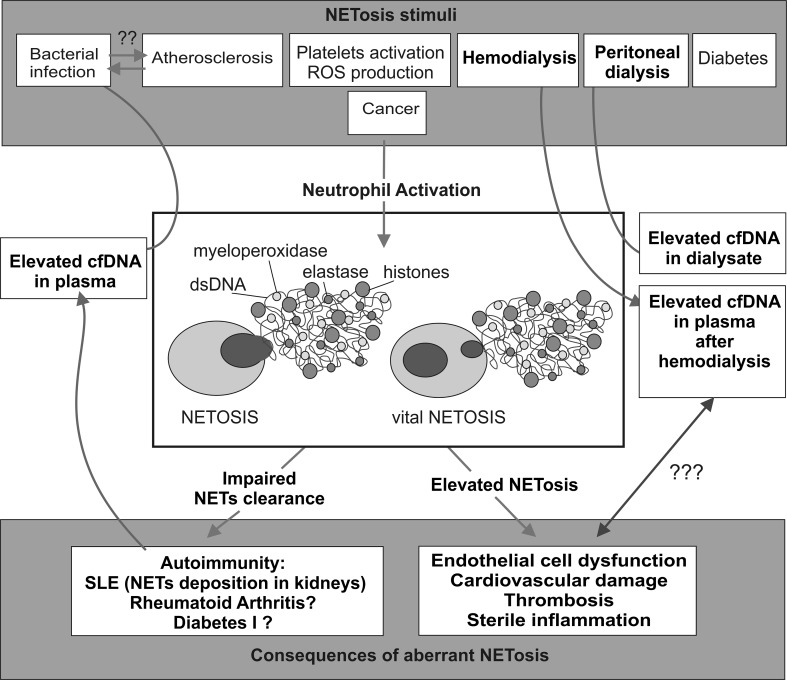
NETosis activation and clearance, clinical conditions, and known laboratory and clinical outcomes

The interconnections among processes of NETosis activation and clearance, clinical conditions, and known laboratory and clinical outcomes are schematically presented in Fig. 1.

It seems that cfDNA if regarded in association with other markers of NETosis may become from an analyte of unknown meaning to an important marker providing not only the information about the reaction of patient’s immune response to hemodialysis. In the group of HD patients studied by us previously, we found rarely the individuals in which plasma cfDNA levels decreased due the process of hemodialysis [14, 47]. It is also necessary to study the inter-individual differences in the NETosis performance with regard to clinical status of HD patients to be able to understand and clarify such observations.

The response to the hemodialytic procedure quantified as elevation of NETosis should be taken into account in the treatment of patients according to the severity of their comorbidities especially diabetes, cardiovascular, and autoimmune diseases.

Further studies focused on the elucidation of mechanisms of NET clearance, and the possible therapeutic interventions allowing the regulation of this process are needed. It is necessary to keep in mind that NETosis functions as a double-edged sword—the balance between correct immune system performance and the potential damaging effects of exacerbated NETosis should be achieved in HD patients with different comorbidities.

The first attempts in this direction have been made. The physiological consequences of NETosis blocking began to be studied [66, 74]. The first study dealing with the levels of cfDNA as markers of NETosis in HD patients appeared [4], but the complicated relationships among the process of hemodialysis, performance of NETosis, and clinical outcomes of HD patients remain to be elucidated in the future. PubMed search focused on the term “NETosis” revealed 234 references but only 13 articles for combination of key words “NETosis” and “Nephrology” in July 2016. It is necessary to follow periodically the development in this interdisciplinary field, because it may bring not only novel insights into the pathogenesis of comorbidities in dialyzed patients, but also clinically useful new diagnostic tests.

## Conclusion

- Elevated plasma levels of cell-free DNA after a hemodialysis procedure may be activated by NETosis which occurs as a consequence of activation of neutrophils during the process of hemodialysis.
- NETs play crucial roles in pathogenesis of numerous comorbidities of HD patients, such as diabetes, cardiovascular and autoimmune diseases.
- Better understanding of NETs formation, clearance, and inter-individual differences in this processes is needed for the development of proper diagnostic and therapeutic approaches.
- The study of plasma cell-free DNA levels together with markers of NETosis could also contribute to evaluation of the influence of hemodialysis on patient’s immune system and to the prediction and management of clinical outcomes with regard to patient’s comorbidities.
